# Increased risk for uterine cancer among first-degree relatives to Swedish gastric cancer patients

**DOI:** 10.1186/s13053-020-00145-y

**Published:** 2020-06-05

**Authors:** Johanna Samola Winnberg, Eva Rudd, Anne Keränen, Kristina Lagerstedt-Robinson, Annika Lindblom, Magnus Nilsson, Mats Lindblad, Krister Sjödahl

**Affiliations:** 1 Division of Surgery, Department of Clinical Science Intervention and Technology (CLINTEC), Karolinska Institutet and Department of Upper Abdominal Diseases, Karolinska University Hospital, Karolinska University Hospital Huddinge, C1:77, 14186 Stockholm, Sweden; 2 Department of Molecular Medicine and Surgery (MMK), Karolinska Institutet, and National Board of Forensic Medicine, Stockholm, Sweden; 3 Division of Pathology, Department of Laboratory Medicine, Karolinska Institutet, and Karolinska University Hospital, Stockholm, Sweden; 4 Department of Molecular Medicine and Surgery (MMK), Karolinska Institutet, and Department of Clinical Genetics, Karolinska University Hospital, Stockholm, Sweden; 5 Division of Surgery, Department of Clinical Science Intervention and Technology (CLINTEC), Karolinska Institutet and Department of Surgery, Norrtälje Hospital, Norrtälje, Sweden

**Keywords:** Gastric cancer, Genetic predisposition to disease, Sweden, Uterine cancer, Neoplastic syndromes, Hereditary

## Abstract

**Purpose:**

In order to further understand genetically predisposing factors of gastric cancer, a retrospective study on 107 patients with gastric cancer was conducted. The family history of cancer cases was registered, in search of associations between gastric cancer and other cancer types.

**Materials and methods:**

Within Stockholm County in Sweden, all patients previously diagnosed with gastric cancer and still alive were invited to participate in the study. Patients were asked to complete a questionnaire about their gastric cancer diagnosis and if any cancers had occurred in their family. A blood sample for DNA extraction was collected. The proportions of different cancer types in the relatives of the patients were compared to the general Swedish population in 1970 and 2010.

**Results:**

Among first- and second-degree relatives to the index patients with gastric cancer, the frequency of uterine cancer as well as gastric cancer was significantly overrepresented compared to the general population in Sweden. The frequency of breast cancer was significantly lower.

**Conclusions:**

There seems to be an increased risk of both gastric cancer and uterine cancer in the families of gastric cancer survivors, indicating a possible hereditary connection between these two cancer types.

## Background

Gastric cancer is a heterogeneous disease, caused by a variety of genetic and environmental predisposing factors. *Helicobacter pylori* is the most well-established risk factor [1]. Tobacco smoking [2–4], dietary factors [5] and low socioeconomic status [6, 7] all predispose to the disease. A family history of gastric cancer is also a strong risk factor [8]. Although most gastric cancers are sporadic, familial aggregation is seen in about 10% of cases [9]. Hereditary cases comprise less than 3% of all gastric cancers [10] and consist of three main autosomal dominant syndromes: hereditary diffuse gastric cancer (HDGC), gastric adenocarcinoma and proximal polyposis of the stomach (GAPPS) and familial intestinal gastric cancer (FIGC) [9].

HDGC was the first of the hereditary gastric cancer syndromes to be recognised, as germline disease causing variants in *CDH1*, coding for E-cadherin, were identified [11]. *CDH1* is located on chromosome 16q22.1. Heterozygous *CDH1* disease causing variants have been described in 18–40% of HDGC families [10]. The International Gastric Cancer Linkage Consortium (IGCLC) defines families with the HDGC syndrome as those fulfilling at least one of following criteria: 1) two or more gastric cancer cases regardless of age, at least one confirmed of histologically diffuse type according to the Laurén classification [12], in first- and second-degree relatives; 2) one case of diffuse gastric cancer < 40 years; 3) personal or family history of diffuse gastric cancer and lobular breast cancer, one diagnosis < 50 years [13]. Not all families fulfilling these criteria have disease causing variants in *CDH1*, indicating that other genes might also be involved in predisposition for diffuse gastric cancer. Germline disease causing variants in two other genes have been described in several unrelated families: *CTNNA1* [14] and *MAP3K6* [15].

GAPPS was defined in 2012 and is characterised by an autosomal dominant transmission of fundic polyposis with no evidence of colorectal or duodenal polyposis or other hereditary gastrointestinal syndromes [16]. The genetic cause has yet to be identified, but recently, it has been suggested that GAPPS could be a variant of Familial Adenomatous Polyposis (FAP) [17]. FIGS, characterised by intestinal histological type gastric cancer [12] with an autosomal dominant inheritance pattern [9], is, on the contrary, practically a selection of families without gastric polyposis. No inherited disease causing variants have been identified so far in this condition.

Gastric cancer risk is also elevated in several other hereditary cancer syndromes, such as Lynch syndrome (disease causing variants in one of the DNA mismatch repair genes), Li-Fraumeni syndrome (*TP53*), familial adenomatous polyposis (*APC*), Peutz-Jeghers syndrome (*STK11*), juvenile polyposis (*SMAD4* or *BMPR1A*) and hereditary breast or ovarian cancer syndrome (*BRCA1* or *BRCA2*) [18].

To further understand genetic predisposing factors of gastric cancer a retrospective study on 107 patients with gastric cancer was conducted. The family history of cancer cases was registered, and pedigrees created, in search for associations between gastric cancer and other cancer types, as well as families interesting for deeper analysis.

## Methods

### Study design and population

A retrospective cohort study on persons diagnosed with gastric cancer in Stockholm County. Information on other cancer diagnoses in the family was collected from persons and familial aggregation of these cancers was estimated. Pedigrees were constructed and, in some persons, further genetic analyses for known gastric cancer genetic syndromes were conducted.

### Data collection

Within Stockholm County in Sweden, all patients previously diagnosed with gastric cancer and still alive were invited to participate in the study. Persons were identified from the Regional Cancer Centre, Stockholm, in august 2013. The Regional Cancer Centre in Sweden administers locally the Swedish Cancer Register, well known for its comprehensive and complete data [19]. Patients were contacted by letter and asked to complete a questionnaire with questions on their gastric cancer diagnosis, any other cancer diagnosis and if any gastric, breast, intestinal, ovarian, uterine, prostate or other cancers had occurred in their family including first- and second-degree relatives. If necessary, interviews per telephone were used to obtain additional information as a complement to the questionnaire. For all relatives with cancer, type of cancer and age at cancer diagnosis were recorded. A written informed consent was given by all participating patients, as well as a signed authorization to collect medical data on index patients, e.g. pathology report of the cancer and date of diagnosis. A blood sample for DNA extraction was collected from all the index patients; blood samples were isolated using a standard protocol at the Department of Clinical Genetics, Karolinska University Hospital.

### Statistical analysis

The distribution of cancer diagnoses in the collected data was evaluated by comparing it to the distribution of cancer diagnoses in the general Swedish population in the years 1970 and 2010. Data on the Swedish population in 1970 and 2010 were obtained from the National Board of Health and Welfare. The population data were assumed to reflect the true distribution, without measurement error. Indirect standardization was used to adjust the data from the Swedish population with regard to sex and age. Age was categorized into five-year intervals. For relatives where data on sex or age were missing, data Missing Completely At Random [20] was assumed. The cancer cases among the index patients’ relatives were assumed to be independent of each other, even where multiple cases were found in the same family. Confidence intervals (CIs) for cancer proportions were calculated for each cancer diagnosis separately, using a binomial distribution. The number of reported subgroups of cancer types were then transformed into proportions, by dividing by the total number of reported cases. A cancer diagnosis was regarded as over- or underrepresented in the relatives of the patients if the reported proportion and its confidence interval was above or below the population reference values for both reference years. Since selection of the material was made on basis of gastric cancer, only diagnoses other than gastric cancer were used in the comparison. The methodology is similar to that used in Wachenfeldt et al. [21], but adjustment with regards to sex and age was made in the present study, and to that used in Wendt et al. [22]. The statistical analysis was performed in R [23].

### Exclusion of known syndromes

Pedigree analyses were done for all the families within the study. For those fulfilling clinical criteria for potential presence of Lynch syndrome according to Bethesda Guidelines [24], we proceeded with immunohistochemistry to evaluate the expression of mismatch repair proteins. Immunohistochemistry (IHC) was performed on 3-μm-thick tissue sections from paraffin-embedded, formalin-fixed tumour. The OptiView DAB IHC Detection kit on the Benchmark ULTRA staining module (Ventana) was used according to the manufacturer’s instructions. Staining with four antibodies MLH1 (M1, Ventana), MSH2 (G219–1129, Ventana), MSH6 (SP93, Ventana) and PMS2 (EPR3947, Ventana) were evaluated. A case was reported as MMR-deficient (dMMR) when displaying total or partial nuclear loss of expression in tumour cells, with retained expression in adjacent normal tissue as a positive control. Expression was reported as MMR proficient (pMMR) when nuclear staining was retained both in tumour cells and positive internal controls. In case of loss of expression of one or more of these proteins, we did further a DNA sequencing analysis on the genes of interest. For those who fulfilled criteria for HDGC according to The International Gastric Cancer Linkage Consortium [13], *CDH1*genetic screening was performed using DNA sequencing.

## Results

### Basic characteristics among index patients

In all, 1091 persons were diagnosed with gastric cancer and registered in Stockholm County during the study period. Of these, 359 gastric cancer patients were still alive and were invited to participate in the study. Some 107 (30%) accepted and were included, out of which 44 (41%) were women and 63 (59%) men. The average age of onset of gastric cancer among the index patients was 63.3 years. Histopathological diffuse type was recorded on 13 (12%) index patients and intestinal type on 93 (87%). In one patient the information about histopathological type was missing.

### Genetic analyses of index patients

Five patients fulfilled criteria for HDGC and thereby underwent analysis for *CDH1* genetic screening. No disease-causing variant was found among these patients. The clinical criteria for potential presence of Lynch syndrome was fulfilled in 23 index patients who underwent further analysis by immunohistochemistry with antibodies against mismatch repair proteins MLH1, MSH2, MSH6 and PMS2. Two patients showed loss of one or more of these proteins and were further analysed with sequencing of DNA. No disease-causing variants were found indicating presence of Lynch syndrome.

### Cancer among first- and second-degree relatives to index patients

In total, the index patients reported 99 cancers among their first-degree relatives alone, out of which 8 (8.08%, CI 3.03–14.14) were uterus cancers. This proportion was significantly higher than identified in the general background population in Sweden 1970 (2.92%) and 2010 (2.58%) respectively (Table 1). A similar overrepresentation of uterus cancer among women was reported, when including information on both first- and second-degree relatives (Table 2).

**Table 1:** Proportion of different cancer types among first degree relatives of both sexes; reported by persons diagnosed with a gastric cancer as compared with expected proportions in background population

	**Reported number (%)**	**Proportion [%]**	**LL 95%**	**UL 95%**	**Proportion [%] in Sweden 1970**	**Proportion [%] in Sweden 2010**	**Reference outside CI**
**All cancer**	**99 (100)**	**100**					
Colon/rectum	18 (18)	18.18	11.11	26.26	12.48	10.9	No
Prostate	13	13.13	7.07	20.2	9.93	17.94	No
Lung and airways	12	12.12	6.06	19.19	7	6.7	No
Stomach	8	8.08	3.03	14.14	7.3	1.43	No
Breast	8	8.08	3.03	14.14	11.92	16.08	No
Uterus	8	8.08	3.03	14.14	2.92	2.58	CI above reference
Kidney and urinary tract excl prostate	5	5.05	1.01	10.1	7.77	6.1	No
Thyroid	4	4.04	1.01	8.08	1.16	1.17	No
Unspecified location	4	4.04	1.01	8.08	3.22	2.11	No
Liver and biliary system	3	3.03	0	7.07	3.12	1.56	No
Ovary and Fallopian tube	3	3.03	0	7.07	3.57	1.51	No
Malignant melanoma	3	3.03	0	7.07	2.09	5.53	No
Blood and lymphatic tissue	3	3.03	0	7.07	7.98	7.65	CI below reference
Cervix	2	2.02	0	5.05	2.96	1.18	No
Brain and nervous system	2	2.02	0	5.05	3.22	2.86	No
Pancreas	1	1.01	0	3.03	3.34	1.76	No
Testicle	1	1.01	0	3.03	0.41	0.56	No
Bone and soft tissue	1	1.01	0	3.03	1.05	0.63	No

Observed cancer cases for first degree relatives of index patients and expected distribution of cases in Sweden from National Board of Health and Welfare (Socialstyrelsen). Expected distribution is adjusted for age and sex in observed cases

**Table 2:** Proportion of different cancer types for first- and second-degree relatives; index women

	**Observed number**	**Proportion [%]**	**LL 95%**	**UL 95%**	**Proportion [%] in Sweden 1970**	**Proportion [%] in Sweden 2010**	**Reference outside CI**
Uterus	9	15.52	6.9	25.86	6.01	5.37	CI above reference
Stomach	8	13.79	5.17	22.41	5.4	1.16	No
Colon/rectum	8	13.79	5.17	22.41	11.84	10.7	No
Breast	8	13.79	5.17	22.41	23.72	32.38	CI below reference
Unspecified location	5	8.62	1.72	17.24	3.48	2.58	No
Ovary and Fallopian tube	4	6.9	1.72	13.79	7.06	3.03	No
Liver and biliary system	3	5.17	0	12.07	3.46	1.53	No
Lung and airways	3	5.17	0	12.07	2.82	6.71	No
Cervix	3	5.17	0	12.07	5.72	2.03	No
Thyroid	2	3.45	0	8.62	1.49	1.59	No
Head and neck	1	1.72	0	5.17	1.59	1.8	No
Kidney and urinary tract excl prostate	1	1.72	0	5.17	5.3	3.75	No
Malignant melanoma	1	1.72	0	5.17	2.42	5.72	No
Bone and soft tissue	1	1.72	0	5.17	1.08	0.61	No
Blood and lymphatic tissue	1	1.72	0	5.17	7.08	6.91	CI below reference

Observed cancer cases for first- and second-degree relatives of index patients and expected distribution of cases in Sweden from National Board of Health and Welfare (Socialstyrelsen). Expected distribution is adjusted for age and sex in observed cases

Index patients reported 180 cancers among their first- and second-degree relatives. The total number of gastric cancers was counted to 25 (13.89%, CI 8.89–18.89) being thereby significantly overrepresented compared to the general cancer population both in 1970 (7.49%) and 2010 (1.43%) (Table 3). The only other significantly higher proportion was found in the group of cancers with an unspecified location.

**Table 3:** Proportion of different cancer types for first- and second-degree relatives; both sexes

	**Observed number**	**Proportion [%]**	**LL 95%**	**UL 95%**	**Proportion [%] in Sweden 1970**	**Proportion [%] in Sweden 2010**	**Reference outside CI**
Colon/rectum	30	16.67	11.67	22.22	12.54	11.16	No
Prostate	26	14.44	9.44	20	9.95	18.07	No
Stomach	25	13.89	8.89	18.89	7.49	1.43	CI above reference
Lung and airways	16	8.89	5	13.33	7.16	6.81	No
Unspecified location	16	8.89	5	13.33	3.3	2.17	CI above reference
Breast	14	7.78	3.89	11.67	11.69	15.83	CI below reference
Uterus	9	5	2.22	8.33	2.93	2.61	No
Liver and biliary system	7	3.89	1.11	6.67	3.14	1.62	No
Kidney and urinary tract excl prostate	6	3.33	1.11	6.11	7.98	6.22	CI below reference
Malignant melanoma	5	2.78	0.56	5.56	1.99	5.46	No
Ovary and Fallopian tube	4	2.22	0.56	4.44	3.44	1.47	No
Thyroid	4	2.22	0.56	4.44	1.04	1.01	No
Blood and lymphatic tissue	4	2.22	0.56	4.44	7.92	7.61	CI below reference
Pancreas	3	1.67	0	3.89	3.43	1.79	No
Cervix	3	1.67	0	3.89	2.78	0.99	No
Brain and nervous system	3	1.67	0	3.89	3.18	2.8	No
Bone and soft tissue	3	1.67	0	3.89	0.96	0.62	No
Head and neck	1	0.56	0	1.67	3.05	2.37	CI below reference
Testicle	1	0.56	0	1.67	0.37	0.55	No

Observed cancer cases for first- and second-degree relatives of index patients and expected distribution of cases in Sweden from National Board of Health and Welfare (Socialstyrelsen). Expected distribution is adjusted for age and sex in observed cases

The proportion of breast cancer among first- and second-degree relatives (7.78%, CI 3.89–11.67) was significantly lower than reported both in 1970 (11.69%) and 2010 (15.83%) (Table 3).

## Discussion

There are some limitations that need to be highlighted. First, our study population was highly selected, which should be considered, when interpreting the results. Only persons that had survived their gastric cancer could be included. Considering the low survival rate of gastric cancer, this represented a selected group of patients. Second, only 30% of the invited patients participated. We do not know the reasons, why patients chose to participate or not to participate in our study as such analysis was not performed. Third, using a questionnaire could introduce bias. Persons completing the form might remember differently than what in fact was true, so called recall bias. Index patients’ cancers were verified but their relatives’ cancers were not verified. In addition, in the questionnaire some cancers were asked for specifically (gastric, breast, ovarian, intestinal, uterine, urinary tract/bladder, cervix, malignant melanoma, thyroid and prostate cancer), while others could only be described under a heading: other cancer. This detail might have affected the results. The specifically named cancers did not, however, share the same results as some were overrepresented and others under- or similarly represented as in the background population. Another weakness of the questionnaire is that we did not specifically ask for the gender of the relatives. Thereby, we could only know the sex of the relatives, if they had a prostate cancer or any of the gynaecologic cancers.

In our study we identified overrepresentation of uterus cancer among relatives to gastric cancer patients. Tzortzatos et al. [25] found a similar association, when they looked at cancer cases among relatives to uterine cancer patients. The proportion of gastric cancer among first- and second-degree relatives, including first cousins, was found to be significantly higher than the expected proportion in Sweden in 1970 and 2010 respectively.

No cases of Lynch syndrome were found among the patients in our study. Thereby, the correlation found between gastric cancer and uterine cancer was seemingly not a part of Lynch syndrome. Thus, there might be an independent connection between the two malignancies.

The average age of onset of gastric cancer among the index patients was 63.3 years, which is rather equivalent to the age of onset of gastric cancer in the general population of Sweden, which is around 65 years of age [26], indicating that the proportion of hereditary gastric cancers was low. Nevertheless, gastric cancer was over-represented in the families of index patients, when both first- and second-degree relatives were included in the analysis. We could not find any disease-causing variant in a known gene correlated to HDGC or Lynch syndrome that would contribute to the apparent familial aggregation, however.

In a previous study of Forsberg et al. [27], significantly more non-colorectal cancers were found among the ones with familial aggregation of colorectal cancers, compared to families with sporadic cases. Among others, significantly more gastric cancers and prostate cancers were observed. We could not find a similar over-representation of either colorectal cancer or prostate cancer among the families included in our study. This might be due to the selection or size of our study population. In fact, we could see that the proportion of colorectal cancer was higher compared to the general cancer population both in 1970 and 2010, both when we looked at first-degree relatives only (Table 1) and when including first- and second-degree relatives (Table 3), but due to wide confidence intervals, the findings were statistically non-significant. The proportion of prostate cancer among male family members was higher compared to the general cancer population both in 1970 and 2010, when we looked at first- and second-degree relatives (Table 4), but also here the result was non-significant.

**Table 4:** Proportion of different cancer types for first- and second-degree relatives; index men

	**Observed number**	**Proportion [%]**	**LL 95%**	**UL 95%**	**Proportion [%] in Sweden 1970**	**Proportion [%] in Sweden 2010**	**Reference outside CI**
Prostate	26	40.62	28.12	53.12	19.39	35.23	No
Stomach	11	17.19	7.81	26.56	9.48	1.69	No
Colon/rectum	9	14.06	6.25	23.44	13.22	11.59	No
Lung and airways	7	10.94	4.69	18.75	11.27	6.9	No
Liver and biliary system	2	3.12	0	7.81	2.84	1.71	No
Kidney and urinary tract excl prostate	2	3.12	0	7.81	10.52	8.56	CI below reference
Unspecified location	2	3.12	0	7.81	3.13	1.77	No
Blood and lymphatic tissue	2	3.12	0	7.81	8.71	8.28	CI below reference
Pancreas	1	1.56	0	4.69	3.88	1.86	No
Testicle	1	1.56	0	4.69	0.73	1.07	No
Brain and nervous system	1	1.56	0	4.69	3.06	2.34	No

Observed cancer cases for first- and second-degree relatives of index patients and expected distribution of cases in Sweden from National Board of Health and Welfare (Socialstyrelsen). Expected distribution is adjusted for age and sex in observed cases

Even though our study has its limitations, it is rather interesting that we can detect an association between gastric cancer and uterine cancer. Currently, we are gathering data for a prospective study with a similar setting, but this time we are including all consecutive cases and thereby patients with both good and poor prognosis. In this follow up study we will hopefully be able to show if the correlation between gastric cancer and uterine cancer, found in this current study, might be correlated with good prognosis in our described patients. Tumour characteristics might also play a key role concerning the connection with uterine cancer.

## Conclusions

The main finding of our study is that gastric and uterine cancer were overrepresented in the families of index patients with gastric cancer. Thereby, there seems to be an association between gastric cancer and uterine cancer. The proportion of breast cancer among the relatives of index patients was significantly lower than expected. Our findings need to be confirmed in future studies.

### Supplementary information

**Additional file 1.**

## Data Availability

The datasets used and analysed during the current study are available from the corresponding author on reasonable request.

## Appendix

**Table 5:** Proportion of different cancer types for first--degree relatives; men

	**Observed number**	**Proportion [%]**	**LL 95%**	**UL 95%**	**Proportion [%] in Sweden 1970**	**Proportion [%] in Sweden 2010**	**Reference outside CI**
Prostate	13	35.14	18.92	51.35	19.86	35.88	No
Colon/rectum	7	18.92	8.11	32.43	13.35	11.49	No
Lung and airways	7	18.92	8.11	32.43	11.27	7.03	No
Stomach	5	13.51	2.7	24.32	9.46	1.72	No
Kidney and urinary tract excl prostate	2	5.41	0	13.51	10.51	8.59	No
Testicle	1	2.7	0	8.11	0.81	1.12	No
Brain and nervous system	1	2.7	0	8.11	2.85	2.23	No
Blood and lymphatic tissue	1	2.7	0	8.11	8.63	8.11	No

Observed cancer cases for first-degree relatives of index patients and expected distribution of cases in Sweden from National Board of Health and Welfare (Socialstyrelsen). Expected distribution is adjusted for age and sex in observed cases

**Table 6:** Proportion of different cancer types for first-degree relatives; women

	**Observed number**	**Proportion [%]**	**LL 95%**	**UL 95%**	**Proportion [%] in Sweden 1970**	**Proportion [%] in Sweden 2010**	**Reference outside CI**
Uterus	8	21.05	7.89	34.21	5.84	5.15	CI above reference
Colon/rectum	5	13.16	2.63	23.68	11.61	10.3	No
Breast	5	13.16	2.63	23.68	23.53	32.07	No
Stomach	3	7.89	0	18.42	5.14	1.15	No
Lung and airways	3	7.89	0	18.42	2.73	6.37	No
Ovary and Fallopian tube	3	7.89	0	18.42	7.14	3.01	No
Unspecified location	3	7.89	0	18.42	3.36	2.49	No
Liver and biliary system	2	5.26	0	13.16	3.35	1.46	No
Cervix	2	5.26	0	13.16	5.92	2.35	No
Thyroid	2	5.26	0	13.16	1.7	1.86	No
Malignant melanoma	1	2.63	0	7.89	2.72	6.08	No
Blood and lymphatic tissue	1	2.63	0	7.89	7.34	7.19	No

Observed cancer cases for first-degree relatives of index patients and expected distribution of cases in Sweden from National Board of Health and Welfare (Socialstyrelsen). Expected distribution is adjusted for age and sex in observed cases

## Abbreviations

HDGC: Hereditary diffuse gastric cancer
GAPPS: Gastric adenocarcinoma and proximal polyposis of the stomach
FIGC: Familial intestinal gastric cancer
IGCLC: The International Gastric Cancer Linkage Consortium
FAP: Familial adenomatous polyposis
CI: Confidence interval
IHC: Immunohistochemistry
MMR: Mismatch repair

## References

[1] Ford AC, Forman D, Hunt R, Yuan Y, Moayyedi P. Helicobacter pylori eradication for the prevention of gastric neoplasia. Cochrane database Syst Rev. 2015;(7):Cd005583. 10.1002/14651858.CD005583.pub2 PMC7263416 26198377

[2] Sjodahl K, Lu Y, Nilsen TI, Ye W, Hveem K, Vatten L (2007). Smoking and alcohol drinking in relation to risk of gastric cancer: a population-based, prospective cohort study. Int J Cancer.

[3] Tredaniel J, Boffetta P, Buiatti E, Saracci R, Hirsch A (1997). Tobacco smoking and gastric cancer: review and meta-analysis. Int J Cancer.

[4] Ladeiras-Lopes R, Pereira AK, Nogueira A, Pinheiro-Torres T, Pinto I, Santos-Pereira R (2008). Smoking and gastric cancer: systematic review and meta-analysis of cohort studies. Cancer Causes Control.

[5] Tsugane S, Sasazuki S (2007). Diet and the risk of gastric cancer: review of epidemiological evidence. Gastric Cancer.

[6] Ji J, Hemminki K (2006). Socio-economic and occupational risk factors for gastric cancer: a cohort study in Sweden. Eur J Cancer Prev.

[7] Kelley JR, Duggan JM (2003). Gastric cancer epidemiology and risk factors. J Clin Epidemiol.

[8] Yaghoobi M, Bijarchi R, Narod SA (2010). Family history and the risk of gastric cancer. Br J Cancer.

[9] Oliveira C, Pinheiro H, Figueiredo J, Seruca R, Carneiro F (2015). Familial gastric cancer: genetic susceptibility, pathology, and implications for management. Lancet Oncol.

[10] van der Post RS, Gullo I, Oliveira C, Tang LH, Grabsch HI, O'Donovan M (2016). Histopathological, molecular, and genetic profile of hereditary diffuse gastric Cancer: current knowledge and challenges for the future. Adv Exp Med Biol.

[11] Guilford P, Hopkins J, Harraway J, McLeod M, McLeod N, Harawira P (1998). E-cadherin germline mutations in familial gastric cancer. Nature..

[12] Laurén PA (1965). The two histological Main types of gastric carcinoma: diffuse and so-called intestinal-type carcinoma. An attempt at a Histo-clinical classification. Acta Pathol Microbiol Scand.

[13] van der Post RS, Vogelaar IP, Carneiro F, Guilford P, Huntsman D, Hoogerbrugge N (2015). Hereditary diffuse gastric cancer: updated clinical guidelines with an emphasis on germline CDH1 mutation carriers. J Med Genet.

[14] Majewski IJ, Kluijt I, Cats A, Scerri TS, de Jong D, Kluin RJ (2013). An alpha-E-catenin (CTNNA1) mutation in hereditary diffuse gastric cancer. J Pathol.

[15] Gaston D, Hansford S, Oliveira C, Nightingale M, Pinheiro H, Macgillivray C (2014). Germline mutations in MAP3K6 are associated with familial gastric cancer. PLoS Genet.

[16] Worthley DL, Phillips KD, Wayte N, Schrader KA, Healey S, Kaurah P (2012). Gastric adenocarcinoma and proximal polyposis of the stomach (GAPPS): a new autosomal dominant syndrome. Gut..

[17] Li J, Woods SL, Healey S, Beesley J, Chen X, Lee JS (2016). Point mutations in exon 1B of APC reveal gastric adenocarcinoma and proximal polyposis of the stomach as a familial adenomatous polyposis variant. Am J Hum Genet.

[18] Petrovchich I, Ford JM (2016). Genetic predisposition to gastric cancer. Semin Oncol.

[19] Regional Cancer Centre Stockholm (2013). Cancerregistret vid Socialstyrelsen.

[20] Rubin DB. Inference and Missing Data 1976:63(3):581–92. 10.2307/2335739.

[21] von Wachenfeldt A, Lindblom A, Gronberg H, Einbeigi Z, Rosenquist R, Gardman C (2007). A hypothesis-generating search for new genetic breast cancer syndromes--a national study in 803 Swedish families. Hereditary Cancer Clin Pract..

[22] Wendt C, Lindblom A, Arver B, von Wachenfeldt A, Margolin S (2015). Tumour spectrum in non-BRCA hereditary breast cancer families in Sweden. Hereditary Cancer Clin Pract.

[23] Development R, Core team. R (2016). A language and environment for statistical computing.

[24] Umar A, Boland CR, Terdiman JP, Syngal S, de la Chapelle A, Ruschoff J (2004). Revised Bethesda guidelines for hereditary nonpolyposis colorectal cancer (lynch syndrome) and microsatellite instability. J Natl Cancer Inst.

[25] Tzortzatos G, Wersall O, Danielsson KG, Lindblom A, Tham E, Mints M (2014). Familial cancer among consecutive uterine cancer patients in Sweden. Hereditary Cancer Clin Pract.

[26] Forsberg A, Keranen A, Vonh S, Picelli S, Papadogiannakis N, Ghazi S (2017). Defining new colorectal Cancer syndromes in a population-based cohort of the disease. Anticancer Res.

[27] Regional Cancer Centre Stockholm. Matstrups- och magsäckscancer 2019 [Available from: https://www.cancercentrum.se/samverkan/cancerdiagnoser/matstrupe-och-magsack.

