# Supplementary material for: Increased risk for uterine cancer among first-degree relatives to Swedish gastric cancer patients
Source: Hered Cancer Clin Pract. 2020 Jun 5;18:12. doi: 10.1186/s13053-020-00145-y (PMC7275318; doi:10.1186/s13053-020-00145-y)
Supplement: Supplementary file 1 — Additional file 1. [file 13053_2020_145_MOESM1_ESM.doc]

EHISTORIA

**FAMILJEHISTORIA sida 1 av 2 Ditt namn: Ditt telefonnummer:**

**Ditt personnummer: Din adress:**

Hur många bröder har du?__________systrar?_____________. Hur många halvsyskon på pappas sida__________/mammas sida?___________

Hur många döttrar har du?__________ söner?_____________. Är dina föräldrar födda i Sverige? Ja Nej

Har någon av dina nära släktingar haft cancer? Om ja, fyll i åldern när de fick sin cancerdiagnos i listan nedan:

*Exempel* Jag Mina Mina Mina Mor Mormor Morfar syskon syskon syskon Far Farmor Farfar syskon syskon syskon till mor till mor till mor

Ventrikelcancer

Bröstcancer

Äggstockscancer

Tarmcancer

Livmodercancer

Urinvägs/blåseca

Livmoderhalscancer

Malignt melanom

Sköldkörtelcancer

Prostatacancer

Annan:

Vilken?:

Ingen cancer

Vet ej

Livmodercancer*

*63*

Bröstcancer

Äggstockscancer

Tarmcancer

Magcancer

Urinblåsecancer

Livmoderhalscancer^¤^

Malignt melanom

Sköldkörtelcancer

Prostatacancer

*50*

Annan cancer

Vilken....................

Ingen cancer

Vet ej

| *endometriecancer  ^¤^ cervixcancer |  |  |  |  |
| --- | --- | --- | --- | --- |

**FAMILJEHISTORIA sida 2 av 2 Ditt personnummer:**

syskon syskon syskon Dina Dina Dina Kusin på Kusin på Kusin på Kusin på

till far till far till far barn barn barn mors sida mors sida fars sida fars sida _______ _______

Ventrikelcancer

Bröstcancer

Äggstockscancer

Tarmcancer

Livmodercancer

Urinvägs/blåseca

Livmoderhalscancer

Malignt melanom

Sköldkörtelcancer

Prostatacancer

Annan:

Vilken?:

Ingen cancer

Vet ej

|  |  |  |  |  |
| --- | --- | --- | --- | --- |

Skriv gärna ned om det finns flera släktingar med cancerdiagnoser här nedan. Ange släktskap, ålder vid cancerdiagnos:
